# A case report of COVID-19 evoked cholangitic liver abscess

**DOI:** 10.1186/s43066-021-00169-6

**Published:** 2022-01-11

**Authors:** Omkolsoum Alhaddad, Maha Elsabaawy, Ahmed Edrees, Essam Elshimy, Dalia Elsabaawy, Tarek Mansour

**Affiliations:** 1 Department Of Hepatology and Gastroenterology, National Liver Institute, Menoufia University, Shebeen El-Kom, 32511 Egypt; 2 Department Of Clinical Pharmacy, National Liver Institute, Menoufia University, Shebeen El-Kom, 32511 Egypt; 3 Department Of Internal Medicine, Faculty of medicine, Ain Shams University, Cairo, Egypt

**Keywords:** COVID-19, Cholangitic, Choledocholithiasis, Stent

## Abstract

**Background:**

Lately, the humanity has been being threatened by the coronavirus disease (COVID-19). The virus-related destructive motives can damage not only the lungs but also the brain, blood vessels, kidneys, and the heart.

**Case presentation:**

A middle-aged female presented with jaundice post-COVID-19 pneumonia. The patient had past history of cholecystectomy 20 years ago. Both laboratory and imaging data revealed a picture of cholestasis with right lobe liver abscess. Despite drainage and culture-based antibiotics, no improvement ensued. Endoscopic retrograde cholangiopancreatography was done revealing mildly dilated common bile duct (CBD), multiple large stones, mildly dilated central biliary radicals, and an old overlooked stent inside the dilated CBD. Papillotomy and papilloplasty were undertaken followed by stones’ extraction with insertion of 2 plastic stents (10 cm× 10 f), and a flow of thick dark bile was inspected. The patient was finally improved and safely discharged.

**Conclusion:**

Herein, we present the first case of long-retained quiescent biliary stent which was over-headed by a cholangitic abscess in the vicinity of COVID pneumonia.

## Background

The emerging COVID-19 disease has been considered this century’s lethal curse [1]. Since the first strenuous apprehension from the Chinese city Wuhan, lots of data has been evolving concerning the novel coronavirus [1]. However, the full-blown clinical scenarios, outcomes, and sequelae have not yet unveiled.

COVID 19-related reports have described a post-recovery state of immunosuppression that would boost serious bacterial and fungal infections [2, 3].

As more than 60% of liver abscesses are of biliary origin, so stones, stents, and biliary anomalies are considered the most conventional predispositions of developing pyogenic liver abscesses [4].

## Case presentation

A 62-year-old lady recently presented to the emergency room of National Liver Institute, Menoufia University, with 3-day history of high fever (41 °C), chills, but neither respiratory symptoms nor alteration of mental status were present. Clinical examination revealed scleral icterus and significant tenderness over the right hypochondrium.

Her history was significant for COVID-19 pneumonia and ICU admission for around 22 days in a nearby hospital 1 month earlier. The patient’s past history was not significant apart from an open cholecystectomy 20 years ago with uneventful postoperative period. The patient is neither diabetic nor hypertensive, and no history of past endoscopic procedures.

Laboratory investigations on presentation showed hyperbilirubinemia, leukocytosis, and pattern of cholestatic hepatitis, but normal COVID-19-related laboratory markers.

**Fig. 1:**
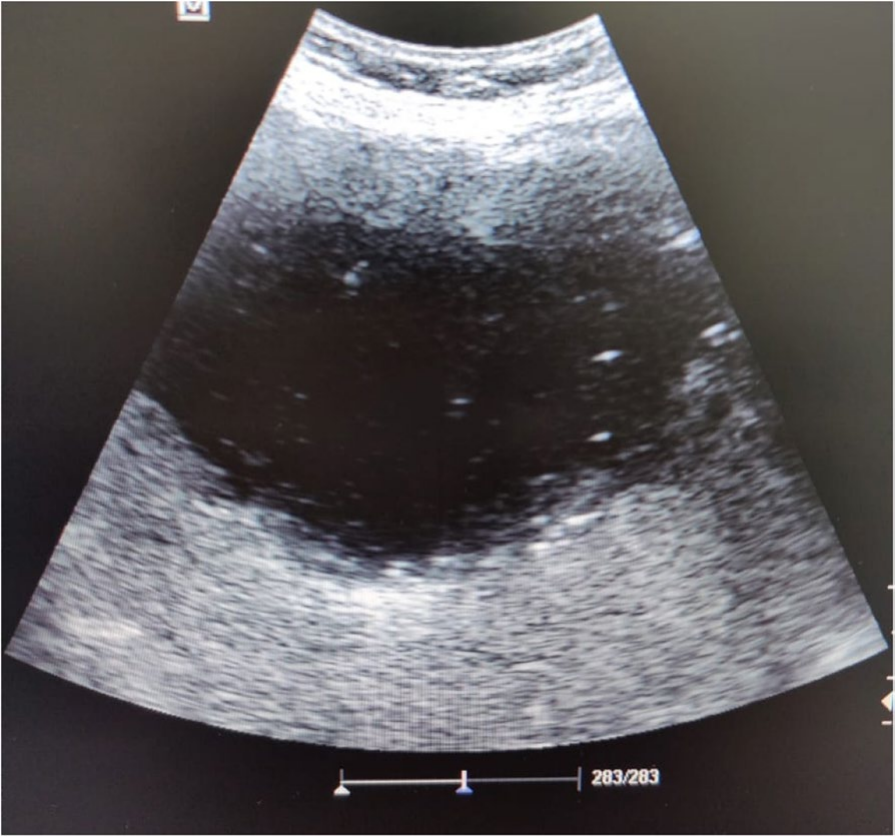
Ultrasonographic picture of the right liver lobe delineating cystic like structure with fine reticulations

**Fig. 2:**
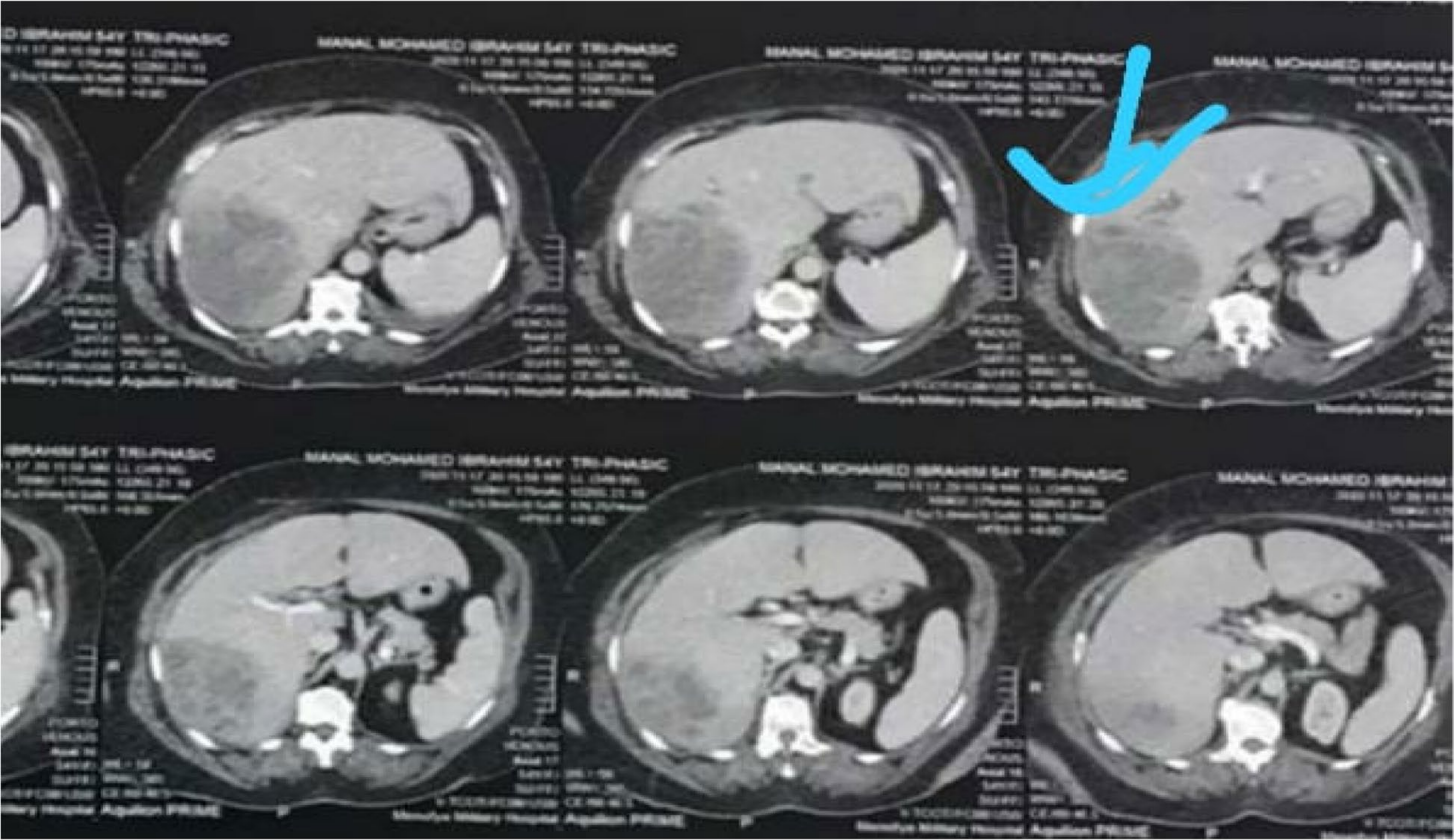
A large right lobe liver abscess with a potential biliary linkage in a biliary-cut

**Fig. 3:**
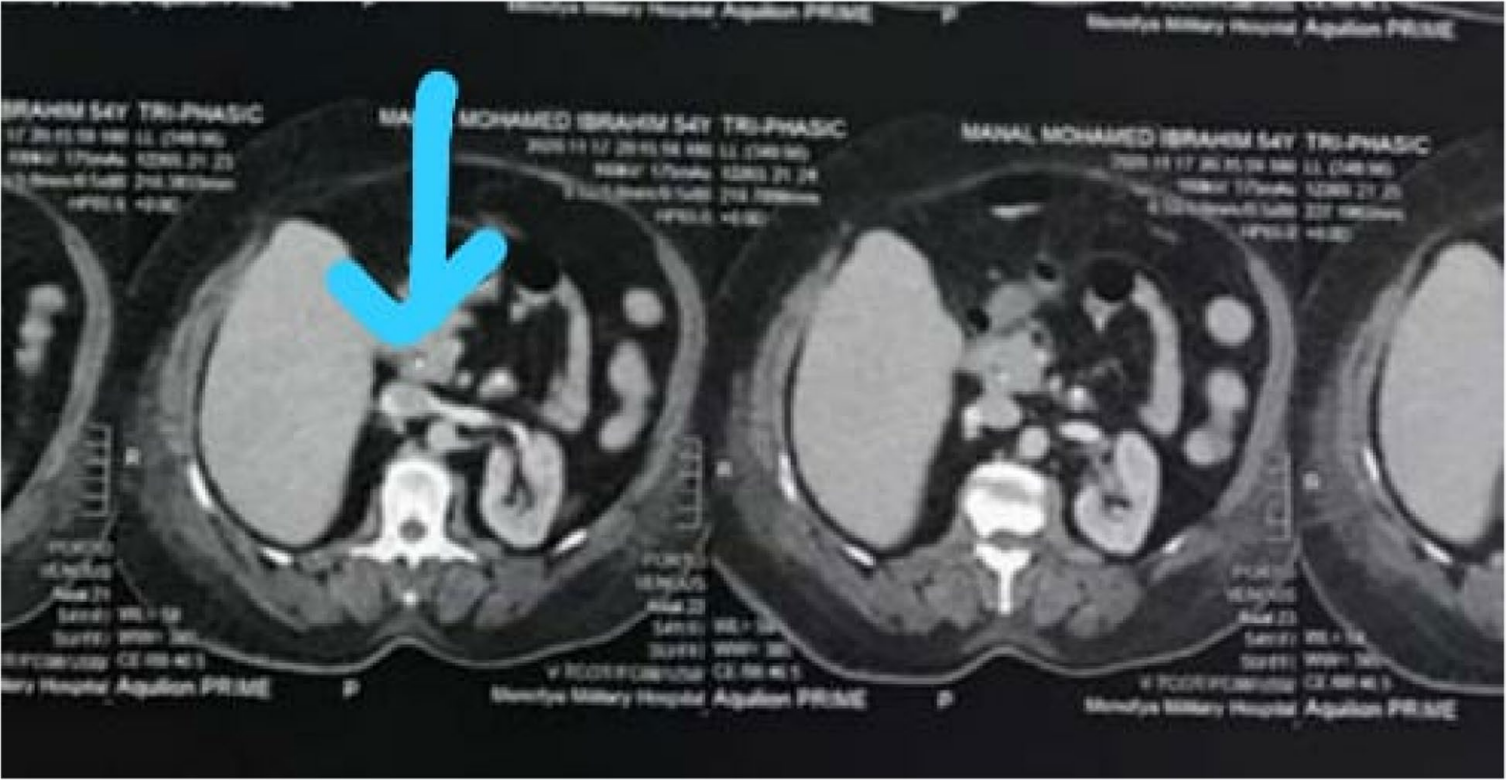
A faint thin stent hardly seen (atypical surgical catheter) on CT images

Also, immediate point of care ultrasound (POCUS) revealed a right hepatic lobe focal lesion measuring (10×10cm), along with an evident stent inside the common bile duct (CBD) (Fig. 1). A consecutive computerized tomography scan of the abdomen confirmed the right lobe focal lesion as a complex abscess occupying large area (Figs. 2 and 3).

Sonography guided, a 10-french pigtail catheter was inserted percutaneously into the abscess cavity meanwhile, with aspiration of 50 mL of purulent fluid that was sent for culture and sensitivity. The patient was transferred to the ward and parenteral broad-spectrum antibiotics were initiated. Few days later, the culture and sensitivity results gave priority to *Escherichia coli*, infection with modulation of prescribed antibiotic.

In the following days, the clinical status of the patient did not show any improvement with persistent fever, discharge from the pigtail, and non-change in sonographic measures of the abscess cavity. The antibiotic regimen was changed according to the results of culture and sensitivity from the pigtail discharge.

Further assessment in the following days has confirmed the condition as non-resolving abscess. This non-response to the classic measures of abscess treatment (culture-based antibiotics and percutaneous drainage) added to the sonographic findings and the cholestatic liver derangement had mandated stepping to endoscopic retrograde cholangiography (ERCP).

**Fig. 4:**
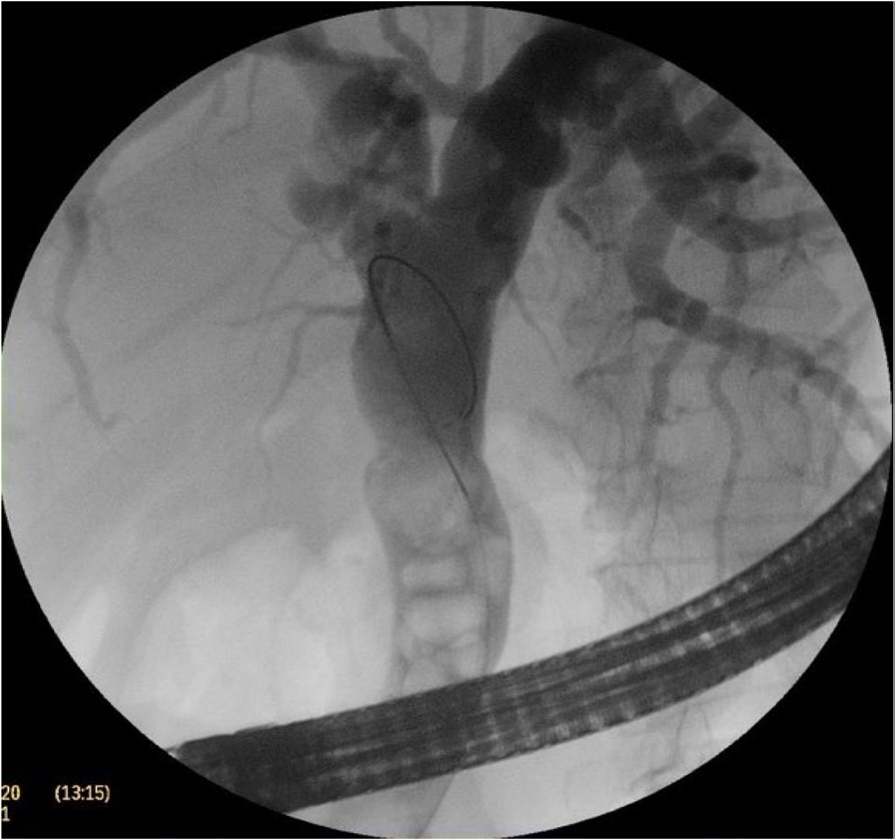
A cholangiogram showing mildly dilated CBD studded with multiple stones

**Fig. 5:**
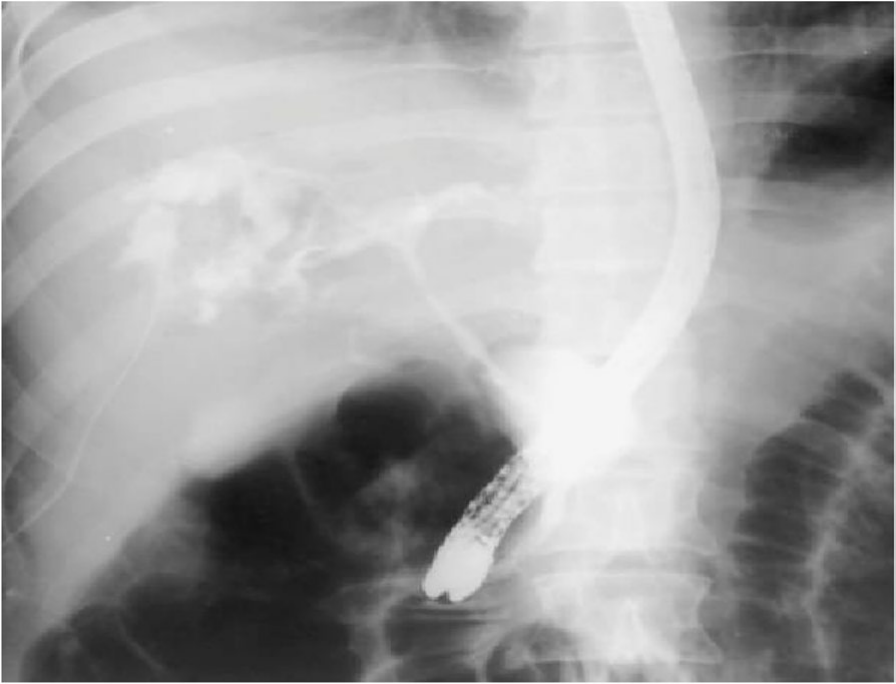
Extravasation of the contrast into the abscess cavity proofing the linkage with the biliary tree

**Fig. 6:**
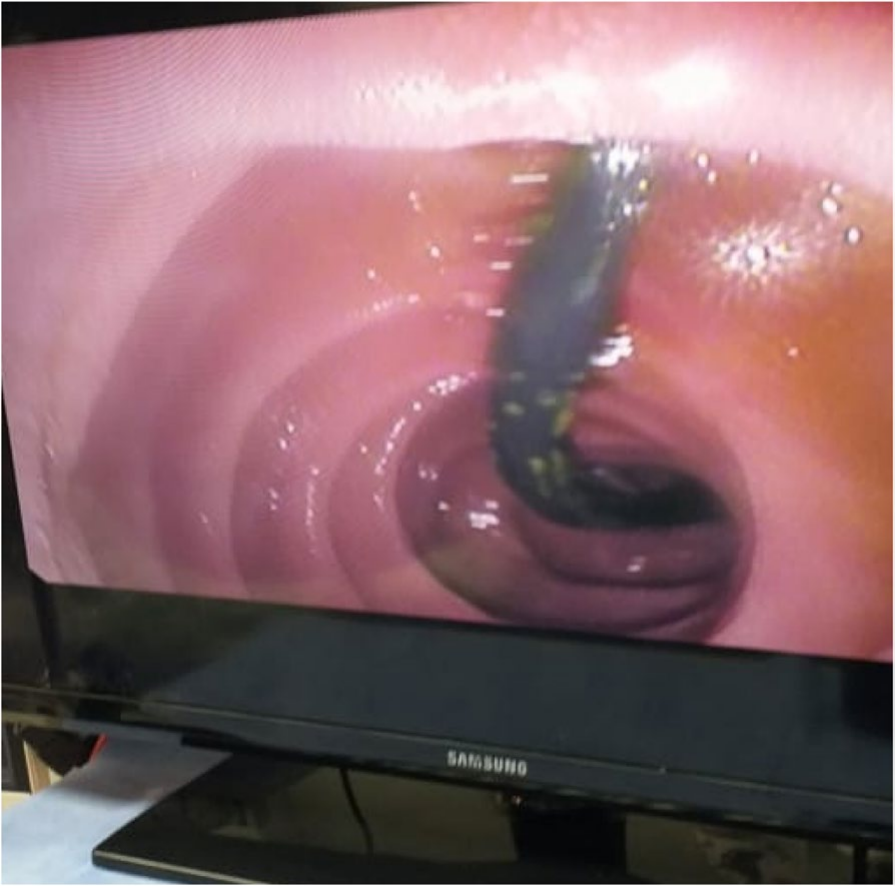
The slipped surgical catheter

The CBD was cannulated after strenuous extraction of a long (20 cm) catheter that was largely displaced into the duodenal lumen. Cholangiogram revealed mildly dilated CBD with multiple large stones accompanied by mildly dilated central biliary radicals. Surprisingly, an old stent was hardly seen inside the dilated CBD, with mild dilatation of the intrahepatic biliary channels (Figs. 4, 5, and 6).

Papillotomy and papilloplasty were undertaken followed by stones’ extraction by a balloon extractor. The procedure was ended by inserting 2 plastic stents (10 cm× 10 f), and a flow of thick dark bile was inspected.

Finally, the patient fully recovered, and the pigtail was removed. Follow-up 2 weeks later revealed stable recovery.

## Discussion

Choledocholithiasis is still considerably observed after cholecystectomy and stones can be either old or newly formed [5]. In the present case, the remote history of cholecystectomy signified the stones on the CBD as de novo choledocholithiasis.

During cholecystectomy and in a case of retained CBD stones, the trans-papillary biliary stenting after CBD compression is considered as an attractive option to avoid T-tube–related complications [6]. Furthermore, it eases clearing the CBD from any calculi during a planned ERCP to retrieve the intraoperatively applied trans-papillary stent [7]. In the present patient, it is clear that the surgically placed stent had been forgotten for 20 years.

Neglected biliary plastic stents may act as a core of matrix for lithogenesis triggered by partial obstruction and slowing of the bile flow [8]. In that milieu, the long-retained biliary stent can foster bacterial proliferation and release of the enzyme beta-glucuronidase, and subsequent precipitation of calcium bilirubinate that then aggregated into stones by an anionic glycoprotein [9].

The initial non-resolution of the abscess despite the pigtail drainage and culture-sensitivity-guided antibiotics then the cholangiographic picture and rapid resolution after adequate biliary drainage, all prove a communication of the abscess with the intrahepatic biliary system.

The mechanism that would explain the post-COVID liver abscess formation in this patient is apparently hard to reach. One speculation could be the immune evasion which perpetually was described as a sequel of SARS-COV2 infection. The virus-mediated immunosuppression enables opportunistic bacteria to colonize vulnerable tissues in the affected patients [10]. The forgotten stent, the partially obstructed biliary system, the new calculi, and the surrounding liver tissue all were providing such vulnerability to post COVID-19 bacterial infection.

In viral pandemics, bacteremia particularly with *Staphylococcus aureus* has been long documented and accused of the associated morbidity and mortality [11]. Severity of the disease and mortality in Spanish flu (1918–1919) and the H1N1 influenza pandemic (2009–2010) are largely attributed to secondary bacteremia [12]. In the recently published reports, staph aureus bacteremia has been documented in patients infected with SARS-CoV-2 [13]. Two reports from New York City have documented bacteremia in patients who suffered from COVID-19. Sepulveda et al. reported that 1.6% of COVID-19 patients had bacteremia, with *S. aureus* accounting for 13% of these bacteremias [14]. Nori et al. reported that 1.9% of COVID-19 patients can develop bacteremias [2].

In recent years, biliary tract disease is the most common source of pyogenic liver abscess [15]. In such a condition, abscesses are usually multiple; however, solitary abscess can occur because of surgical manipulation or indwelling biliary stents [16].

Thus, another speculation in the presented case is that COVID-19 pneumonia-associated systemic bacteremia along with hematogenous dissemination can be the background pathology behind bacterial cholangitis and abscess formation.

*E. coli* is the prevalent liver abscesses pathogen and had been incriminated in triggering right lobe solitary abscesses [17, 18].

COVID-19 had been convicted in many liver derangements starting from just elevated liver enzymes up to acute fulminant liver failure supporting the hepatic injurious nature of the virus [19, 20]. A condition might be the clue of this case, as the ongoing COVID-19 hepatic injury paved the way to be the bed for *E. coli* proliferation and invasion in an immunocompromised patient with an overlooked biliary stent.

The presenting case is exceptional for the asymptomatic de novo choledocholithiasis projecting over the 20-year forgotten surgically applied trans-papillary stent. Also, the post-COVID cholangitic abscess was over-heading a quiescent biliary disease.

## Conclusion

In this case, the unique sequel of COVID-19 appraises a new aspect for that threatening virus. More importantly, it necessitates careful evaluation of COVID-19 affected patients, and a full eye follow-up after recovery.

## Data Availability

All the data is available in the manuscript.

## Abbreviations

CBD: Common bile duct
ERCP: Endoscopic retrograde cholangiopancreatography
